# *Caryophylli* Cortex Suppress PD-L1 Expression in Cancer Cells and Potentiates Anti-Tumor Immunity in a Humanized PD-1/PD-L1 Knock-In MC-38 Colon Cancer Mouse Model

**DOI:** 10.3390/nu16244415

**Published:** 2024-12-23

**Authors:** Aeyung Kim, Eun-Ji Lee, Jung Ho Han, Hwan-Suck Chung

**Affiliations:** Korean Medicine (KM) Application Center, Korea Institute of Oriental Medicine, Daegu 41062, Republic of Korea; jistr@kiom.re.kr (E.-J.L.); hanjh1013@kiom.re.kr (J.H.H.)

**Keywords:** *Caryophylli* cortex, immune checkpoint, programmed death 1, programmed death ligand 1, anticancer activity

## Abstract

Background/Objectives: Immune checkpoints are essential for regulating excessive autoimmune responses and maintaining immune homeostasis. However, in the tumor microenvironment, these checkpoints can lead to cytotoxic T cell exhaustion, allowing cancer cells to evade immune surveillance and promote tumor progression. The expression of programmed death-ligand 1 (PD-L1) in cancer cells is associated with poor prognoses, reduced survival rates, and lower responses to therapies. Consequently, downregulating PD-L1 expression has become a key strategy in developing immune checkpoint inhibitors (ICIs). *Caryophylli* cortex (CC), derived from the bark of the clove tree *Syzygium aromaticum*, possesses antioxidant and cytotoxic properties against cancer cells, yet its potential as an ICI remains unclear. Methods: In this study, we aimed to investigate whether CC extract modulates PD-L1 expression in cancer cells and activates T cell immunity through a co-culture system of cancer cells and T cells, as well as in hPD-L1/MC-38 tumor-bearing animal models. Results: Our findings indicate that CC extract significantly downregulated both constitutive and inducible PD-L1 expression at non-toxic concentrations for cancer cells while simultaneously enhancing cancer cell mortality and T cell activity in the co-culture system. Furthermore, the administration of CC extract to hPD-L1/MC-38 tumor-bearing mice resulted in a greater than 70% reduction in tumor growth and increased infiltration of CD8+ T cells within the tumor microenvironment. Principal component analysis identified bergenin, chlorogenic acid, and ellagic acid as active ICIs. Conclusions: These findings suggest that CC extract exerts a potent antitumor effect as an immune checkpoint blocker by inhibiting PD-L1 expression in cancer cells and disrupting the PD-1/PD-L1 interaction.

## 1. Introduction

The capacity of tumor cells to evade the immune response, a phenomenon referred to as immune escape, and their acquired resistance to anti-cancer therapies are major challenges to the effective management of cancer [1]. A fundamental mechanism underlying this process is the interaction between Programmed Death Ligand 1 (PD-L1) on cancer cells and the Programmed Death 1 (PD-1) receptor on cytotoxic T lymphocytes (CTLs), inhibiting T cell activity [2,3,4]. The PD-1/PD-L1 axis is one of several immune checkpoint regulators that play critical roles in maintaining self-tolerance and modulating the duration and intensity of immune responses, primarily by inhibiting adaptive T cell responses. Tumor cells exploit this mechanism to suppress anti-tumor responses, leading to CTL exhaustion and heightened resistance to pro-apoptotic signals. Currently, immune checkpoint blockade (ICB) using anti-PD-L1 and anti-PD-1 antibodies is a promising therapeutic strategy for cancer patients [5]. However, approximately 30% of patients relapse, highlighting the need for alternative strategies to regulate the PD-1/PD-L1 axis [6].

PD-L1 is markedly overexpressed in melanoma, lung, ovarian, pancreatic, colorectal, and breast cancers. In breast cancer, elevated PD-L1 levels are associated with unfavorable clinical outcomes and correlated with negative prognostic indicators, such as an increased proliferation index, larger tumor size, the absence of estrogen or progesterone receptors, HER2 positivity, and higher tumor grade [7,8,9,10]. These associations suggest that the inhibition of PD-L1 expression is a promising approach for immune checkpoint-based cancer therapies. Natural extracts and their derivatives exhibit low toxicity and minimal side effects while demonstrating pharmacological activities that effectively manage various conditions, including cancer, hypertension, diabetes, inflammation, and aging. The pharmacological and nutritional advantages associated with these ingredients have resulted in an increasing interest in the development of natural medicines and functional health foods. This trend is further evidenced by the corresponding growth of the natural product market. Recent studies have demonstrated that compounds derived from natural extracts such as berberine, luteolin, apigenin, and cosmosiin have the potential to reactivate anti-cancer immune responses and improve therapeutic efficacy by modulating either constitutive or inducible PD-L1 expression in cancer cells [11,12,13,14].

The clove tree, *Syzygium aromaticum,* is an evergreen tree in the family Myrtaceae that is cultivated in tropical regions in India, Indonesia, Malaysia, and Sri Lanka. Its flower buds have been esteemed for centuries for their applications in food preservation, flavoring, perfumery, and traditional medicine [15,16]. Clove flower buds exhibit anti-inflammatory, anti-diabetic, antimicrobial, antiviral, antioxidant, and hepatoprotective activity [17,18]. Ethanol extracts of clove bark, known as *Caryophylli* cortex (CC), mitigate diabetes-induced renal damage by inhibiting advanced glycation end-product (AGE)-induced glucotoxicity and oxidative stress [19]. Furthermore, methanol extracts of CC are cytotoxic to MCF-7 breast cancer cells [20]. However, the potential of CC as an immune checkpoint inhibitor (ICI) to enhance anti-tumor immune responses remains unexplored. Therefore, this study examined whether water (WCC) and ethanol (ECC) extracts of CC enhance anti-cancer efficacy by reducing PD-L1 expression on cancer cells and activating T cell immunity, disrupting PD-L1-mediated immune tolerance.

## 2. Materials and Methods

### 2.1. WCC and ECC Preparation

Lyophilized powder of WCC (Cat. No. KOC-DW-335) and ECC (Cat. No. KOC-70E-261) were obtained from KOC Biotech (Daejeon, Republic of Korea) and dissolved in 100% dimethyl sulfoxide (DMSO, #D8418, Sigma Chemical Co., St. Louis, MO, USA) to achieve a final concentration of 100 mg/mL. After filtering through a 0.22-μm disk filter, aliquots were kept at −20 °C.

### 2.2. Cell Lines

The MDA-MB-231 (human breast adenocarcinoma cell line, HTB-26), DLD-1 (human colon adenocarcinoma cell line, CCL-221), and HEK-293 (human embryonic kidney cell line, CRL-1573) cells were obtained from the American Type Culture Collection (ATCC, Manassas, VA, USA). Human PD-L1-expressing MC38 cells (hPD-L1/MC38), derived from C57BL/6 murine colon adenocarcinoma cells, were purchased from the Shanghai Model Organisms Center (Shanghai, China). Jurkat T cells expressing the firefly luciferase gene under the control of nuclear factor of activated T cells (NFAT) response elements, with constitutive expression of human PD-1 (NFAT/PD-1/Jurkat T cells), were obtained from BPS Bioscience (San Diego, CA, USA). MDA-MB-231, hPD-L1/MC-38, and HEK-293 cells were cultured in Dulbecco’s Modified Eagle Medium with 4.5 g/L glucose (DMEM), while DLD-1 and NFAT/PD-1/Jurkat T cells were cultured in RPMI 1640 medium. Both culture media were supplemented with 10% heat-inactivated fetal bovine serum (FBS) and 100 IU penicillin/100 μg/mL streptomycin (P/S). The cells were cultured at 37 °C in a humidified incubator with 5% CO_2_. DMEM, RPMI, FBS, and P/S were obtained from Thermo Fisher Scientific (Waltham, MA, USA).

### 2.3. Chemicals and Antibodies

Crystal violet solution (1% aqueous solution, #V5265), collagenase IV, DNase, and trypsin inhibitor were all obtained from Sigma-Aldrich (St. Louis, MO, USA). CellTracker Green 5-chloromethylfluorescein diacetate (CMFDA, #C7025) dye and recombinant human interferon-γ (IFN-γ, #300-02) were obtained from Thermo Fisher Scientific (Waltham, MA, USA). APC-conjugated anti-human CD274 (B7-H1, PD-L1, #393610) antibody was purchased from BioLegend (San Diego, CA, USA). Antibodies against β-actin (#4970) and PD-L1 (#15165) were sourced from Cell Signaling Technology (Beverly, MA, USA).

### 2.4. Cell Viability Assay

To determine non-cytotoxic concentrations of WCC and ECC, cell viability was measured using Cell Counting Kit-8 (#CK04, Dojindo Molecular Technologies, Inc., Rockville, MD, USA), according to the manufacturer’s protocol. In brief, cells (5 × 10^3^/well) were seeded in a 96-well plate, grown overnight, and treated with increasing doses of WCC, ECC, or vehicle (0.1% DMSO) for 24 h. Following the removal of the culture supernatants, the cell viability was measured with a SpectraMax3 microplate reader (Molecular Devices, LLC, Sunnyvale, CA, USA).

### 2.5. Analysis of Membrane PD-L1 Expression Using Flow Cytometry and Immunoblotting

To investigate the effects of WCC and ECC on PD-L1 expression in cancer cells, MDA-MB231, which are known for their high constitutive PD-L1 expression [21], and hPD-L1/MC38 cells were exposed to specified concentrations of WCC and ECC for 24 h. In the case of DLD-1 cells, WCC and ECC were pre-treated for 12 h and then stimulated with 20 ng/mL IFN-γ for 24 h. For flow cytometry analysis, cells were collected, rinsed with wash buffer (2% FBS in PBS), and incubated with APC-conjugated anti-PD-L1 for 30 min at 4 °C. Following washing, fluorescence levels were quantitatively assessed using a Gallios flow cytometer with Kaluza Flow Analysis software (Beckman Coulter, Inc., Brea, CA, USA). For immunoblotting, cells were harvested, washed with cold PBS, and lysed in RIPA buffer with a protease inhibitor cocktail. After spinning at 13,000 rpm for 15 min at 4 °C, cell lysates were collected, and protein concentration was measured using the bicinchoninic acid assay (#23227; Thermo Fisher Scientific). Proteins (25 μg per lane) were separated on an SDS-polyacrylamide gel and transferred to an Immobilon-P PVDF membrane (Millipore, Bedford, MA, USA). The membrane was incubated with EzBlock Chemi Solution (ATTO Korea, Daejeon, Republic of Korea) for 1 h at 25 °C and then exposed to a specific primary antibody (1:1000 dilution) overnight at 4 °C. After washing with TBS-T (0.1% Tween), membranes were incubated with an HRP-conjugated secondary antibody (1:4000 dilution) for 1 h at 25 °C. Target proteins were visualized with the ImageQuant LAS4000 mini (GE Healthcare, Piscataway, NJ, USA) and SuperSignal West Femto Maximum Sensitivity Substrate (Thermo Fisher Scientific). Protein levels were quantified with ImageJ 1.54f software, and the relative intensities of the immunoblot bands were normalized to the β-actin value.

### 2.6. Co-Culture Experiments for T Cell-Mediated Tumor Cell Killing Assay

MDA-MB231 cells were seeded at a density of 2 × 10^5^ cells per well in 24-well culture plates and exposed to different concentrations of WCC and ECC for 24 h. Following the removal of the culture medium, the cells underwent three washes with PBS and were subsequently stained with CellTracker CMFDA dye (2.5 μM) for 30 min at 37 °C. After two additional washes with PBS, NFAT/PD-1/Jurkat T cells were added to the MDA-MB231 cells labeled with the green dye and incubated for 24–48 h. Subsequently, the PD-1/Jurkat cells were eliminated, and the remaining MDA-MB231 cells were examined using a fluorescence microscope. To assess cell viability, the cells were subjected to crystal violet staining. Following a wash with distilled water, the stained cells were dissolved in a 1% SDS solution, and then the absorbance was measured at 590 nm using a SpectraMax3 microplate reader.

### 2.7. Experimental Mice and Allograft Tumor Model

Mice with humanized PD-1, developed on the C57BL/6J genetic background and genetically modified to express the complete human PD-1 protein (referred to as hPD-1/C57BL/6J), were obtained from the Shanghai Model Organisms Center in China. To establish the humanized PD-1/PD-L1 MC38 tumor allograft model, hPD-L1/MC38 cells (3 × 10^5^ in 200 μL PBS) were injected subcutaneously on the right flank of each hPD-1/C57BL/6J mouse. Tumor growth was monitored, and size was measured using digital calipers (Hi-Tech Diamond, Westmont, IL, USA). Mice were raised in a controlled environment at the KIOM for research purposes. The experiments followed the Institutional Animal Care and Use Committee (IACUC) guidelines at the Korea Institute of Oriental Medicine (KIOM) and were approved under number KIOM-D-21-091.

### 2.8. Isolation of Tumor Infiltrating CD8+ T Cells and Co-Culture with hPD-L1/MC38 Cells

To isolate tumor-infiltrating T cells (TILs), tumor masses from hPD-L1/MC-38 tumors in hPD-1 mice were removed, cut into small pieces, and then digested in RPMI media containing collagenase IV (1 mg/mL), DNase (0.1 mg/mL), and trypsin inhibitor (1 mg/mL) for 40 min at 37 °C. The digested cells were then filtered through mesh cell strainers (SPL Life Sciences, Pocheon, Republic of Korea) of decreasing sizes (100-μm, 70-μm, and 40-μm) to obtain a single-cell suspension. CD8+ TILs were isolated using immunomagnetic separation with a Mouse CD8+ T cell isolation kit (Miltenyi Biotec, Waltham, MA, USA) and then activated with CD3/CD28 magnetic Dynabeads (Life Technologies, Carlsbad, CA, USA) for 24 h at 37 °C. Simultaneously, hPD-L1/MC38 cells were treated with different concentrations of WCC and ECC for 24 h. After washing, the activated CD8+ TILs (Effector cells, E) were added to the WCC- or ECC-treated hPD-L1/MC38 cells (Target cells, T) at a ratio of E:T = 5:1. The cells were then incubated for 48 h, and the viable hPD-L1/MC38 cells were identified after crystal violet staining. Quantitation was performed using a SpectraMax3 microplate reader as described above.

### 2.9. NFAT Luciferase Activity Assay

T cell activity in NFAT/PD-1/Jurkat T cells co-cultured with MDA-MB231 or HEK-293 cells was evaluated using the Luciferase Assay System (#E4550, Promega, Madison, WI, USA) per the manufacturer’s protocol. In brief, following co-culture with cancer cells, floating NFAT/PD-1/Jurkat T cells were harvested and subjected to centrifugation at 1200 rpm for 10 min. Subsequently, the cells were lysed using luciferase cell culture lysis buffer (#E1531, Promega). The cell lysates were then mixed with luciferase assay reagent in a 1:5 ratio, and luminescence was measured immediately using a SpectraMax Luminometer (Molecular Devices).

### 2.10. Enzyme-Linked Immunosorbent Assay (ELISA)

The concentrations of IL-2 and granzyme B, which are released by activated T cells, were quantified using ELISA following the manufacturer’s instructions. The BD OptEIATM Human IL-2 ELISA Set (#555190, BD Biosciences, San Diego, CA, USA), human Granzyme B SimpleStep ELISA Kit (ab235635, Abcam, Cambridge, MA, USA), BD OptEIATM Mouse IL-2 ELISA Set (#555148, BD Biosciences), and Granzyme B Mouse ELISA Kit (#88-8022, Thermo Fisher Scientific) were utilized for analyzing the co-culture supernatants or mouse sera.

### 2.11. Assay for Blocking the Interaction Between PD-1 and PD-L1

To examine the effects of WCC and ECC on the PD-1 and PD-L1 interaction, competitive ELISA was performed using the PD-1: PD-L1 Inhibitor Screening Assay Kit (#72005, BPS Bioscience Inc., San Diego, CA, USA) according to the manufacturer’s instructions. In brief, recombinant hPD-L1 (#71104, BPS Bioscience) was immobilized on 96-well plates at a concentration of 1 µg/mL (100 µL per well) and incubated overnight at 4 °C. Plates were washed three times with PBS-T (0.05% Tween), blocked for 1 h at 25 °C with PBS-T containing 2% bovine serum albumin, and washed again. Various concentrations of WCC and ECC were added, followed by a 1-h incubation at 25 °C. Biotinylated hPD-1 (#71109, BPS Bioscience) was put into the wells and incubated for 2 h at 25 °C. After washing three times with PBS-T, 100 µL of HRP-conjugated streptavidin was added to each well. Following a 1-h incubation, the plates underwent three washes with PBS-T. The relative chemiluminescence was quantified using an ECL solution and a SpectraMax L Luminometer. For the positive control, an anti-PD-1 neutralizing antibody (α-PD-1, #71120, BPS Bioscience) was used.

### 2.12. In Vivo Anti-Tumor Activity of WCC in Humanized PD-1/PD-L1 Mouse Model

To create tumor masses, hPD-L1/MC38 cells (3 × 10^5^ cells suspended in 200 μL of PBS) were injected under the skin on the right side of each hPD-1/C57BL/6J mouse. Tumor growth was measured twice weekly with a digital caliper. Once the tumor volume reached 100–150 mm^3^ on day 12, the mice were randomly assigned to one of four groups (n = 5 per group): a Normal group (no tumor + PBS), a Control group (tumor + PBS), and WCC groups receiving either a low or high dose of WCC (tumor + 100 or 300 mg/kg WCC). The mice were orally administered either the PBS or WCC daily for 15 days. Tumor volume and body weight were recorded on days 1, 4, 8, 11, 14, and 16. Tumor volume was calculated using the formula: Tumor volume = (*a*^2^ × *b*) × 0.52, where a and b represent the shortest and the longest diameters of the tumors, respectively. After the tumors were removed and weighed, the tumor suppression rate (TSR) was determined using the formula: TSR (%) = [(*Vc* − *Vt*)/*Vc*] × 100, where *Vc* represents the tumor volume of the control group and *Vt* represents the tumor volume of the WCC treatment group.

### 2.13. Immunohistochemistry

Tumors were fixed in a 10% formalin solution, dehydrated, and embedded in paraffin. The paraffin block was then sliced into consecutive 10-μm sections, which were placed on a microscope slide. To conduct immunostaining, the sections were deparaffinized, rehydrated, subjected to epitope retrieval, and had peroxidase activity blocked. Subsequently, the sections were treated with antibodies targeting CD8 (#98941) and granzyme B (#46901) overnight and then visualized using a DAKO EnVision Kit (DAKO, Jena, Germany). Following counterstaining with Mayer’s hematoxylin, the images were examined using an Olympus BX53 microscope and an XC10 digital camera (Tokyo, Japan).

### 2.14. Statistical Analysis

Data analysis was performed with GraphPad Prism 9.5.1 (GraphPad Software, San Diego, CA, USA). Results are expressed as means ± standard error of the mean (SEM) from multiple trials. Variations between groups were assessed using one-way analysis of variance (ANOVA) with Dunnett’s multiple comparison test, and statistical significance was set at *p* < 0.05 (* *p* < 0.05, ** *p* < 0.01, and *** *p* < 0.001). All experiments, except in vivo studies, were repeated at least three times.

## 3. Results

### 3.1. WCC and ECC Decrease PD-L1 Expression in Cancer Cells

**Figure 1:**
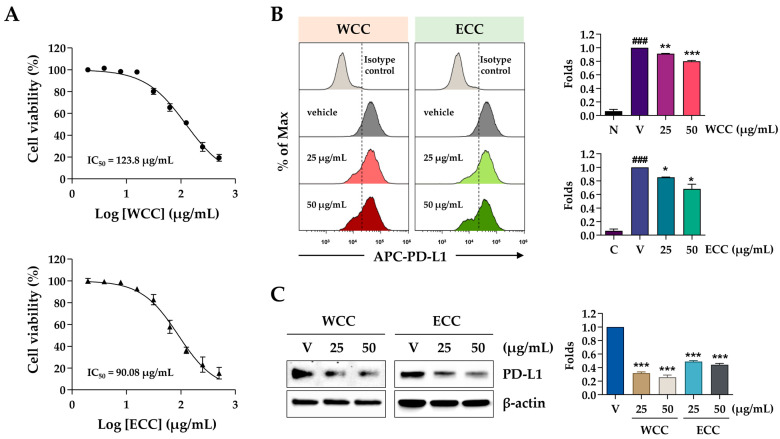

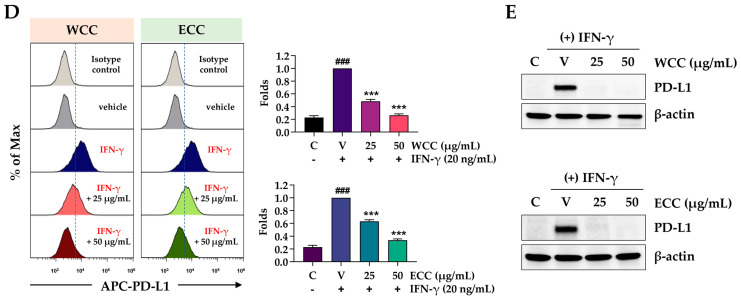
Effects of WCC and ECC on the PD-L1 expression in cancer cells. (**A**) Following the treatment of MDA-MB-231 cells with increasing concentrations of WCC and ECC for 24 h, relative cell viability was assessed and expressed as the means ± SEM (n = 3). (**B**) MDA-MB231 cells were treated with 25 and 50 μg/mL of WCC and ECC for 24 h, and then the levels of membrane PD-L1 were detected by flow cytometry. Relative fluorescence intensity was reported as the means ± SEM (n = 3). (**C**) After treatment with 25 and 50 μg/mL of WCC and ECC for 24 h, PD-L1 expression in MDA-MB231 cells was analyzed by immunoblotting. Relative band intensities after normalization to β-actin expression were presented as the means ± SEM (n = 3). (**D**) DLD-1 cells were pretreated with WCC and ECC (25 and 50 μg/mL) for 6 h and then stimulated with 20 ng/mL IFN-γ for 24 h. Membrane PD-L1 was detected by flow cytometry, with relative intensity compared to IFN-γ/vehicle-treated cells shown as the means ± SEM (n = 6). ### *p* < 0.001 vs. isotype control, * *p* < 0.05, ** *p* < 0.01, *** *p* < 0.001 vs. -treated cells. (**E**) DLD-1 cells pretreated with WCC and ECC were stimulated with IFN-γ for 40 h. Whole cell lysates were analyzed for the PD-L1 expression by immunoblotting.

First, to exclude the cytotoxic effects of WCC and ECC, we treated MDA-MB231 cells with these extracts for 24 h and assessed cell viability. The half-maximal inhibitory concentrations (IC_50_) of WCC and ECC were 123.8 and 90.08 μg/mL, respectively (Figure 1A). Accordingly, non-toxic concentrations of 25 and 50 μg/mL were chosen for subsequent experiments. After treating MDA-MB231 cells with vehicle (0.1% DMSO), WCC, or ECC for 24 h, flow cytometry was used to verify PD-L1 expression on the cell membrane. As shown in Figure 1B, the fluorescence intensity of PD-L1 decreased in a dose-dependent manner with WCC and ECC treatment. Furthermore, the expression of PD-L1 protein in MDA-MB231 cells decreased significantly in whole-cell lysates after treatment with WCC and ECC (Figure 1C). As PD-L1 in cancer cells can be upregulated by pro-inflammatory cytokines such as IFN-γ [12,22], we next examined whether WCC and ECC regulate inducible PD-L1 expression in IFN-γ-stimulated DLD-1 cells. After IFN-γ stimulation, flow cytometry showed a notable rise in PD-L1 expression in DLD-1 cells, and inducible PD-L1 expression decreased significantly with WCC and ECC treatment (Figure 1D). We also found that PD-L1 protein levels upregulated by IFN-γ were almost completely suppressed by WCC and ECC (Figure 1E). These results indicate that WCC and ECC effectively reduce constitutive or inducible PD-L1 expression in cancer cells.

### 3.2. Interaction Between PD-L1 and PD-1 Impairs T Cell Function

**Figure 2:**
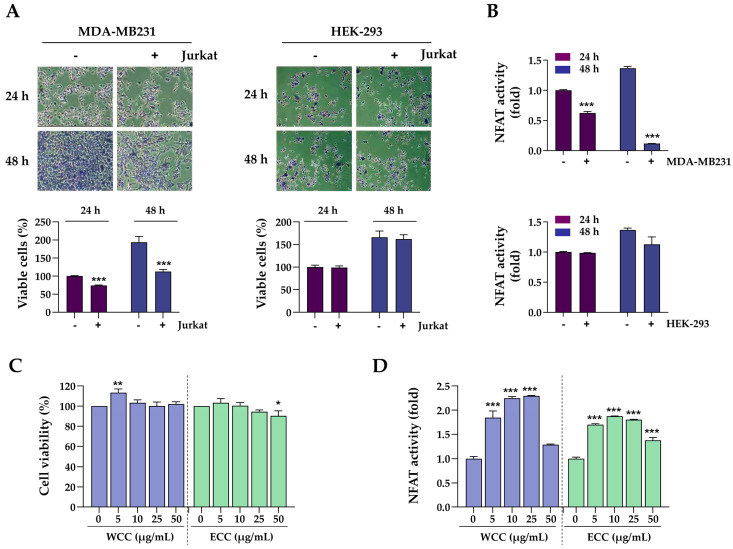
Effects of PD-1/PD-L1 interaction on the cell viability of cancer cells and the NFAT activity of T cells. (**A**) MDA-MB231 and HEK-293 cells were seeded on culture plates, incubated overnight, and then co-cultured with NFAT/PD-1/Jurkat T (Jurkat) cells at a ratio of 1:10. After 24 and 48 h, Jurkat cells were removed, and the remaining MDA-MB231 and HEK-293 cells were stained with crystal violet solution to quantify viable cells. Original magnification ×100. *** *p* < 0.001 vs. w/o Jurkat cells. (**B**) After co-culturing with MDA-MB231 and HEK-293 cells for 24 h, NFAT activity in Jurkat cells was assessed. *** *p* < 0.001 vs. w/o MDA-MB231 cells. (**C**) Jurkat cells were treated with WCC and ECC for 24 h, and cell viability was assessed using a CCK assay. (**D**) After treating Jurkat cells with WCC and ECC for 24 h, NFAT activity was assessed. Data are presented as the means ± SEM (n = 3). * *p* < 0.05, ** *p* < 0.01, *** *p* < 0.001 vs. vehicle-treated cells.

In co-culture experiments, the interaction between PD-L1 in cancer cells and PD-1 in T cells leads to T cell apoptosis and inactivation, resulting in tolerance of the cancer cells [23]. To investigate the effect of PD-L1 expression on T cell function in co-culture experiments, we used MDA-MB231 cells, which exhibit high expression levels of PD-L1, alongside PD-L1-deficient HEK293 cells. As shown in Figure 2A, after 24 and 48 h of co-culture with PD-1-expressing T cells, the viability of MDA-MB231 cells decreased by 25% and 42%, respectively, compared to the control. HEK-293 cells showed minimal changes in cell viability. Concurrently, NFAT activity in Jurkat cells was assessed to validate T cell functionality after co-culture. There was a 38% decrease after 24 h of co-culture with MDA-MB231 and a 91% decrease after 48 h. Following co-culture with HEK-293 cells, there was minimal reduction in NFAT activity (Figure 2B). These findings suggest that reducing PD-L1 expression in cancer cells through WCC and ECC treatment may enhance the effectiveness of anti-cancer treatments by inhibiting the PD-1/PD-L1 immune checkpoint and maintaining T cell function. Next, we explored the effects of WCC and ECC on T cell proliferation and activity. The results showed a slight increase in cell viability at low WCC concentrations, with no cytotoxic effects observed within the experimental range (Figure 2C). NFAT activity increased by 2.3- and 1.8-fold with 25 μg/mL WCC and ECC, respectively (Figure 2D). Based on these findings, to investigate the specific effects of WCC and ECC on PD-L1 regulation in cancer cells without directly affecting T cells, cancer cells were pre-treated with WCC and ECC, which were washed out, and then the cancer cells were co-cultured with T cells.

### 3.3. WCC and ECC Improve T Cell-Mediated Killing of MDA-MB231 Cells and Enhance the Immune Response

**Figure 3:**
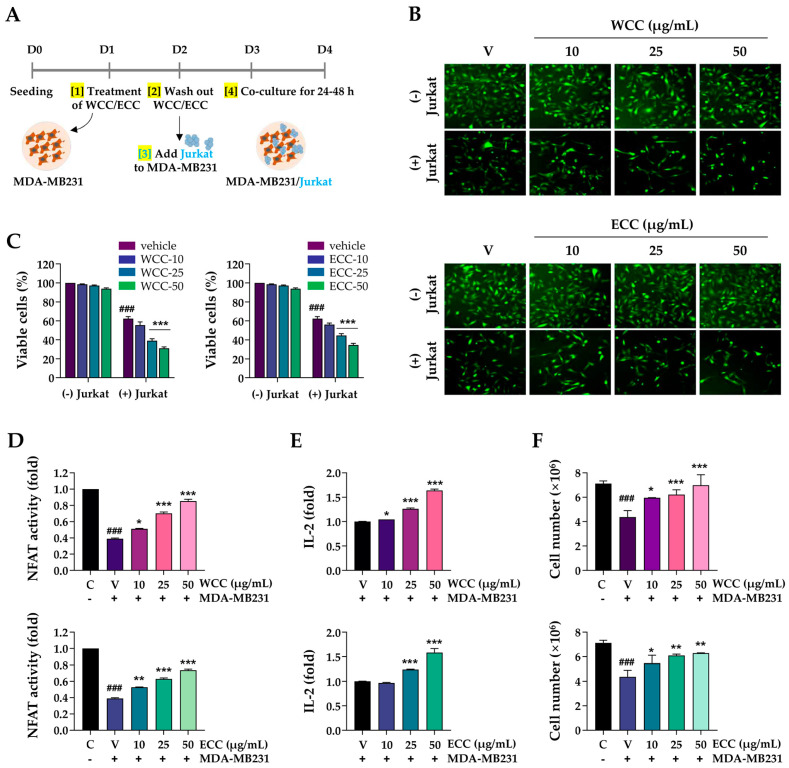
Impact of WCC and ECC on the cancer cells and T cells in co-culture. (**A**) Experimental scheme of cancer cells and T cell co-culture. (**B**) MDA-MB231 cells labeled with green dye were treated with WCC and ECC for 24 h, then co-cultured with or without Jurkat cells for another 24 h. Subsequently, the Jurkat cells were removed, and the remaining cancer cells were examined under a fluorescence microscope. Original magnification ×100. (**C**) Remaining cancer cells were stained with crystal violet solution and quantified after co-culture. (**D**) After co-culture, the NFAT activity of Jurkat cells was measured. (**E**) IL-2 levels in culture supernatants were measured by ELISA. (**F**) After co-culture, Jurkat cells were harvested, and viable cells were counted. Data are presented as the means ± SEM (n = 3). ### *p* < 0.001 vs. control cells, * *p* < 0.05, ** *p* < 0.01, *** *p* < 0.001 vs. vehicle-treated cells.

PD-L1 in cancer cells facilitates their evasion of the immune system. In light of the findings that WCC and ECC diminish PD-L1 expression in cancer cells, we investigated the implications of this reduction on T cell-mediated cytotoxicity against cancer cells and the overall anti-tumor immune response within co-culture systems. After treating MDA-MB231 cells with WCC and ECC for 24 h, the cells were washed, and then Jurkat cells were added to the MDA-MB231 cells at a ratio of 1:5 and co-cultured for up to 48 h (Figure 3A). In the absence of Jurkat cells, WCC and ECC had minimal impact on the viability of MDA-MB231 cells. However, when co-cultured with Jurkat cells with WCC- and ECC-treated MDA-MB-231 cells, green fluorescence-labeled MDA-MB231 cells decreased in a manner correlated with reduced PD-L1 expression (Figure 3B). Viable cells were quantified using crystal violet staining, which showed that WCC and ECC significantly improved the ability of Jurkat cells to kill MDA-MB231 cells (Figure 3C and Supplementary Figure S1). Furthermore, NFAT activity in Jurkat cells, which was reduced by MDA-MB231 cells, increased significantly with WCC and ECC in a concentration-dependent manner. Specifically, treatment with 50 μg/mL WCC and ECC increased NFAT activity by 2.2- and 1.92-fold, respectively, compared to the untreated control, and restored NFAT activity to 75–85% of the level in Jurkat cells that had not been exposed to MDA-MB231 cells (Figure 3D). ELISA assays using medium from MDA-MB231 and Jurkat cell co-cultures revealed that WCC and ECC significantly increased IL-2 levels, suggesting positive effects on T cell activity (Figure 3E). Next, Jurkat cells were counted after co-culture with MDA-MB231 cells. When co-cultured with WCC- and ECC-treated MDA-MB231 cells, Jurkat cell death was suppressed, leading to an increase in cell numbers due to reduced PD-L1 expression (Figure 3F). Collectively, our findings indicate that WCC and ECC suppress PD-L1 expression on cancer cells, thereby reducing PD-L1/PD-1 immune checkpoint-mediated immune suppression, which in turn enhances T cell-mediated cytotoxicity against cancer.

### 3.4. WCC and ECC Enhance the Ex Vivo Anti-Cancer Activity of Tumor Infiltrating CD8+ T Cells Against MC-38 Cells

**Figure 4:**
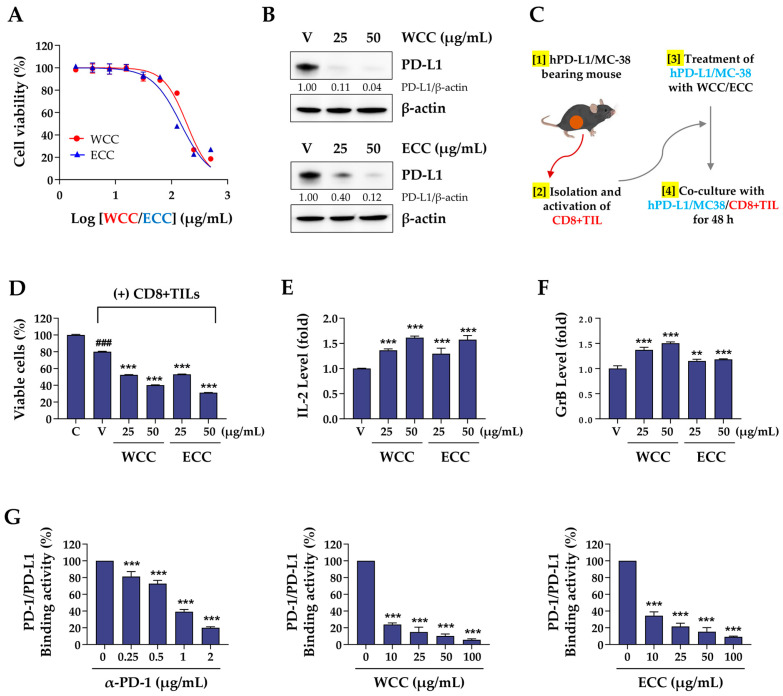
Effects of WCC and ECC on the anti-cancer activity of tumor-specific CD8+ T cells in a co-culture setting. (**A**) After treating hPD-L1/MC-38 cells with WCC and ECC for 24 h, cell viability was examined by CCK assay. (**B**) The levels of PD-L1 protein in hPD-L1/MC-38 cells treated with WCC and ECC were assessed using western blotting. Relative values were calculated after normalizing to β-actin. (**C**) Experimental design of co-culture with hPD-L1/MC38 cells and CD8+ TIL cells. (**D**) After co-culturing with CD8+ TILs, the viability of the remaining cancer cells was assessed. (**E**,**F**) Following co-culture, IL-2 (**E**) and granzyme B (GrB) (**F**) levels in the supernatants were measured by ELISA. (**G**) PD-1/PD-L1 binding activity with or without WCC and ECC was determined by competitive ELISA. Anti-PD-1 antibody was used as a positive control. Data are presented as the means ± SEM (n = 3). ### *p* < 0.001 vs. control cells, ** *p* < 0.01, *** *p* < 0.001 vs. vehicle-treated cells.

Before testing the effects of WCC and ECC in a humanized PD-1/hPD-L1 MC-38 cancer mouse model, we confirmed their efficacy in ex vivo experiments. The viability of hPD-L1/MC-38 cells was assessed after treatment with WCC and ECC, yielding IC_50_ values of 187.3 and 146.2 μg/mL, respectively (Figure 4A). In immunoblotting analysis, WCC and ECC reduced PD-L1 expression in hPD-L1/MC-38 cells by nearly 90%, as expected (Figure 4B). To generate cancer-specific cytotoxic CD8+ T cells, hPD-L1/MC38 cancer cells were inoculated into hPD-1 mice, and then tumor-infiltrating CD8+ T cells (CD8+ TIL) were isolated from the tumor mass (Figure 4C). In co-culture, these CD8+ TILs had approximately 23% cytotoxicity against control hPD-L1/MC38 cells but showed significantly improved cytotoxicity of 50–70% against WCC- and ECC-treated hPD-L1/MC38 cells (Figure 4D and Supplementary Figure S2). When co-cultured with WCC- and ECC-treated hPD-L1/MC38 cells, the levels of IL-2 and GrB, which are T cell activation indicators, increased significantly in the medium (Figure 4F). A competitive PD-1/PD-L1 ELISA was conducted to verify whether WCC and ECC could inhibit the interaction between PD-1 and PD-L1. Similar to the anti-PD-1 antibody’s effect as a positive control, WCC and ECC inhibited the PD-1 and PD-L1 interaction in a dose-dependent manner. The effect of WCC was slightly stronger than that of ECC, with both producing over 90% inhibition at 100 μg/mL (Figure 4G). This finding suggests that administering WCC and ECC to mice with hPD-L1/MC-38 tumors can inhibit the PD-1/PD-L1 immune checkpoint, leading to strong anti-cancer immune responses.

### 3.5. WCC and ECC Suppress hPD-L1/MC-38 Tumor Growth in Humanized PD-1 Mice

**Figure 5:**
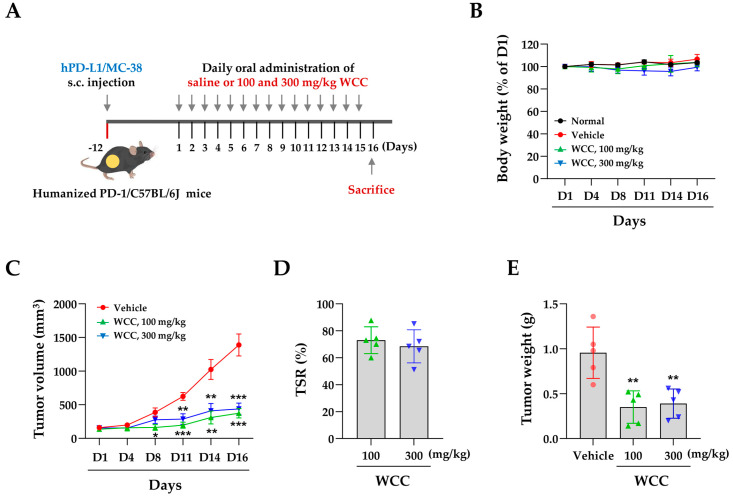
Effects of oral administration of WCC on the growth of hPD-L1/MC-38 tumors in humanized PD-1 mice. (**A**) Experimental scheme for tumor injection, administration of WCC, and sacrifice. (**B**) Body weight changes compared to day 1 (D1) in normal and tumor-bearing mice were measured on days 4, 8, 11, 14, and 16 after WCC administration. (**C**) The size of the hPD-L1/MC-38 tumor mass was measured on days 4, 8, 11, 14, and 16 following the administration of either WCC or vehicle. (**D**) Tumor suppression rate by WCC administration was compared to control mice given a vehicle. (**E**) On day 16, mice were euthanized, and their tumors were removed and weighed. Data are presented as the means ± SEM (n = 5). * *p* < 0.05, ** *p* < 0.01, *** *p* < 0.001 vs. vehicle-treatment.

**Figure 6:**
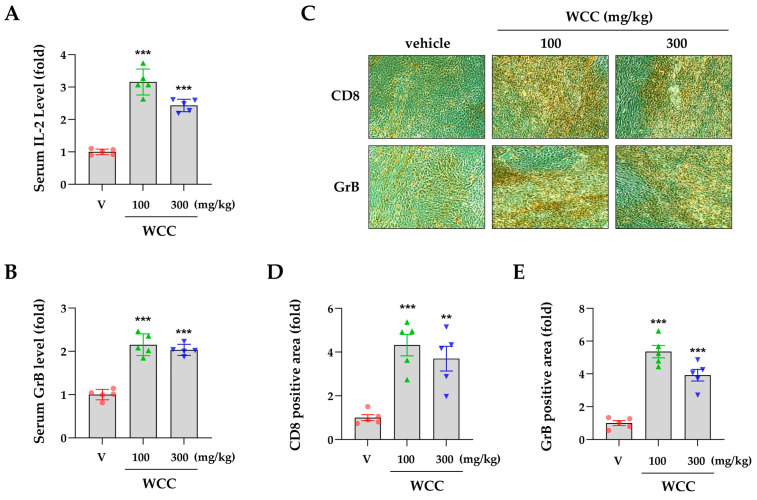
Effects of oral administration of WCC on the T cell activity in humanized PD-1 mice. (**A**,**B**) On day 16, serum from mice in each group was isolated, and the levels of IL-2 (**A**) and granzyme B (GrB) (**B**) were determined using ELISA. (**C**) Representative images of CD8- and GrB-positive areas within tumors from mice administered with WCC or vehicle. Original magnification ×200. (**D**,**E**) The relative areas positive for CD8 and granzyme B (GrB) within the tumors of the WCC-treated group were compared to those of the vehicle-treated group. Data are expressed as the means ± SEM (n = 5). ** *p* < 0.01, *** *p* < 0.001 vs. vehicle-treatment.

To investigate the impact of WCC on in vivo tumorigenesis, we established a colorectal cancer allograft model with hPD-L1/MC-38 cells in hPD-1/C57BL/6J mice. Tumor-bearing mice were randomly divided into three groups and given either control saline or 100 or 300 mg/kg WCC orally daily for 15 days (Figure 5A). Normal mice without tumors were also given saline. During the experiment, the administration of WCC did not result in weight loss, and no abnormal behavior or movements were observed (Figure 5B). By contrast, tumor growth in mice administered WCC was consistently suppressed from day 8 until the end of the experiment, compared to control mice administered saline (Figure 5C and Supplementary Figure S3A). Finally, WCC administration significantly reduced hPD-L1/MC-38 tumor growth, with suppression rates of 73.03% at 100 mg/kg and 68.48% at 300 mg/kg (Figure 5D). The tumor-suppressive effects of WCC were further demonstrated by comparing tumor weight (Figure 5E) and observing tumors ex vivo (Supplementary Figure S3B). To investigate tumor suppression mechanisms and T cell activity in anti-cancer responses after WCC administration, mouse serum was collected and the IL-2 and GrB levels were determined. The IL-2 and GrB levels in mice given WCC were approximately three and two times higher, respectively, than those in saline-treated control mice (Figure 6A,B). In histochemical analysis, there was a notable increase in the infiltration of CD8+ T cells into hPD-L1/MC-38 tumors in mice treated with WCC. GrB levels were significantly elevated in the tumor tissues of these mice (Figure 6C–E and Supplementary Figure S4). Overall, our results suggest that WCC exhibits potent anti-cancer effects by lowering the PD-L1 level of tumors, which in turn suppresses the PD-1/PD-L1 interaction and boosts the effectiveness of anti-cancer CD8+ T cells.

### 3.6. Chlorogenic Acid Reduces PD-L1 Expression and Enhances T Cell-Mediated Anti-Tumor Activity

**Figure 7:**
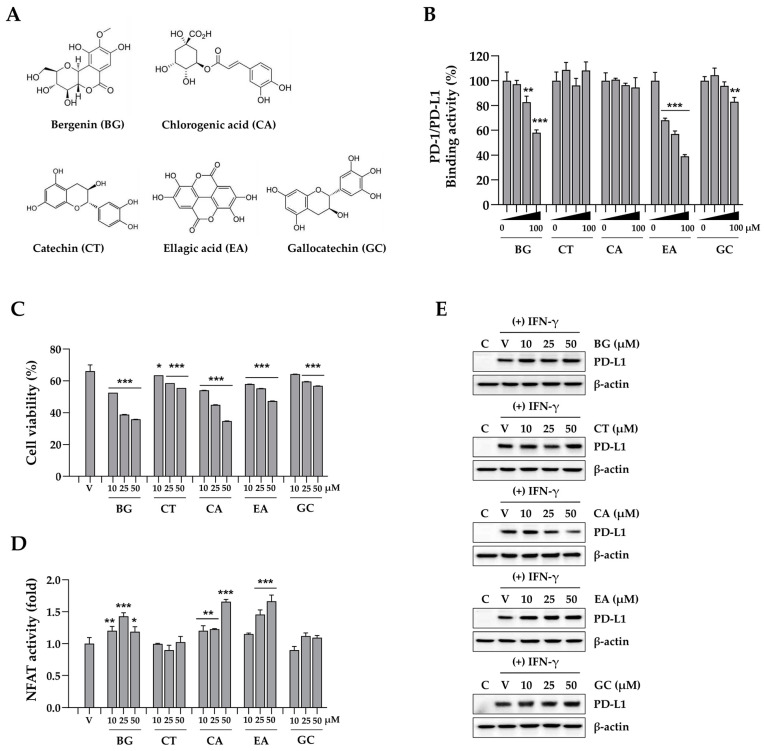
Effects of compounds in *Caryophylli* Cortex extract on the PD-1/PD-L1 axis. (**A**) The chemical structure of bergenin (BG), catechin (CT), chlorogenic acid (CA), ellagic acid (EA), and gallocatechin (GC). (**B**) The binding activity of PD-1/PD-L1 in the presence of BG, CT, CA, EA, and GC was evaluated using a competitive ELISA. (**C**) MDA-MB231 cells were pre-treated with each compound for 24 h and then co-cultured with Jurkat cells. After 24 h, Jurkat cells were removed, and the remaining MDA-MB231 cells were quantified. (**D**) Following co-culture, the NFAT activity of Jurkat cells was measured. * *p* < 0.05, ** *p* < 0.01, *** *p* < 0.001 vs. vehicle-treated cells. (**E**) The levels of IFN-γ-inducible PD-L1 protein in DLD-1 cells were analyzed via western blotting.

To identify the active constituents acting as PD-1/PD-L1 ICIs in CC extract, five components were analyzed: bergenin (BG), catechin (CA), chlorogenic acid (CA), ellagic acid (EA), and gallocatechin (GC) (Figure 7A). In a competitive ELISA examining PD-1/PD-L1 binding, BG and EA efficiently blocked the interaction between PD-1 and PD-L1 in a dose-dependent manner, whereas CT, CA, and GC did not (Figure 7B). After testing their cytotoxicity toward MDA-MB231 cells, no cytotoxicity was found for BG, CT, CA, or GC up to 100 μM. However, EA decreased cell viability by approximately 15% at 100 μM (Supplementary Figure S5A). Based on this result, MDA-MB231 cells and Jurkat cells were co-cultured at a maximum EA concentration of 50 μM. After co-culture with Jurkat cells, the surviving MDA-MB-231 cells were stained with crystal violet and quantified. Viability decreased in a concentration-dependent manner with BG, CA, and EA, whereas CT and GC had minimal effects at 50 µM (Figure 7C and Supplementary Figure S5B). In addition, NFAT activity in Jurkat cells co-cultured with BG-, CA-, and EA-treated MDA-MB-231 cells exceeded that in vehicle-treated cells (Figure 7D). To evaluate PD-L1 expression, DLD-1 cells were pretreated for 24 h and stimulated with IFN-γ. CA significantly reduced PD-L1 expression in a concentration-dependent manner, while other treatments had no effect (Figure 7E). These findings indicate that BG and EA enhance anti-cancer activity by inhibiting PD-1 binding to PD-L1, while CA regulates PD-L1 expression as an ICI.

## 4. Discussion

ICIs play a pivotal role in the management of malignant tumors, augmenting therapeutic strategies and enhancing cancer patient survival rates [24]. The PD-1/PD-L1 signaling pathway is an important target in the therapeutic management of various malignancies, as PD-L1 is expressed across a diverse range of tumor types and in immune cells present within the tumor microenvironment. Blockade of the PD-1/PD-L1 interaction facilitates the targeting of cancer cells by cytotoxic T lymphocytes. The inhibition of this interaction can result in significant antitumor responses [1,2,23]. As its levels are correlated with patient responsiveness, making it an effective predictive biomarker, regulating PD-L1 expression is vital in ICI therapy [4,8]. Elevated PD-L1 expression frequently signifies a more immunosuppressive microenvironment, which may increase susceptibility to immune checkpoint blockade. Current research focuses on the regulatory pathways of PD-L1 expression, influenced by inflammatory cytokines, genetic alterations, epigenetic modifications, oncogenic signaling, and oxidative stress. Strategies aimed at modulating PD-L1 and integrating therapies with other immune checkpoints are currently under investigation to improve the efficacy of ICIs and broaden patient eligibility. To assess the efficacy of an ICI, it is crucial to distinguish its therapeutic effects from cytotoxic effects exerted on cancer cells. For example, berberine has been shown to exhibit anti-cancer properties via mechanisms such as the inhibition of cell proliferation, induction of cell cycle arrest, and promotion of either apoptosis or autophagy across various cancer types [25,26]. Notably, at concentrations that do not elicit cytotoxic effects in cancer cells, berberine enhances the sensitivity of these cells to co-cultured T cells by downregulating PD-L1 expression on their surface [14]. Furthermore, berberine showed antitumor effects in Lewis tumor xenograft models by augmenting the immune response of tumor-infiltrating cells [27].

Reactive oxygen species (ROS) exhibit a dual role within the tumor microenvironment, promoting tumor growth while also inducing apoptosis in cancer cells when present in excessive amounts. Therefore, the regulation of ROS is essential for the effectiveness of anti-cancer therapies. ROS can upregulate or downregulate the expression of PD-L1 in cancer cells, frequently exhibiting a consistent trend in their regulatory effects. For example, arsenic trioxide was found to elevate both ROS levels and PD-L1 expression. In contrast, an extract from *Anoectochilus formosanus*, which exhibits free radical scavenging activity, demonstrated an opposing effect, resulting in a decrease in both ROS levels and PD-L1 expression. Furthermore, epigallocatechin gallate, recognized for its potent antioxidant properties, decreased PD-L1 expression in lung cancer cells, which is associated with the restoration of T cell activity [28]. These findings suggest that the antioxidant-mediated suppression of PD-L1 may enhance anticancer immune responses, highlighting its potential as an ICI.

Previously, we demonstrated that extracts of *Salvia plebeia*, *Oenothera biennis*, and Korean red ginseng inhibit the binding of PD-1 to PD-L1, thereby showing significant anti-cancer effects in humanized PD-1/PD-L1 knock-in mouse models of colorectal cancer [29,30,31]. Furthermore, we validated the synergistic anti-cancer effects of combination therapy with established colorectal cancer treatments, such as oxaliplatin and pembrolizumab, combined with herbal extracts, such as unripe *Rubus coreanus* and *Sanguisorba radix* [32,33]. Our recent study found that cosmosiin, a natural flavone glycoside, boosts T cell antitumor activity and induces cancer cell apoptosis by inhibiting PD-L1 expression, suggesting its potential as an ICI [12].

In this study, we investigated the potential of CC extract as an ICI to enhance anti-cancer immunity and induce apoptosis in cancer cells. To mitigate the potential for a direct cytotoxic effect of CC extract on cancer cells, as previously documented [20], we initially assessed a non-toxic concentration of the CC extract on these cells. Thereafter, all subsequent experiments were conducted using this non-toxic concentration for the treatment of cancer cells. We showed that both WCC and ECC significantly reduced the expression of both constitutive and inducible PD-L1 at non-toxic concentrations (Figure 1). When administered to T cells, these extracts effectively enhanced immune activity by increasing NFAT activity. In co-culture experiments involving PD-L1-expressing cancer cells and PD-1-expressing Jurkat T cells, approximately 50% of the Jurkat T cells were eliminated due to interactions between PD-1 and PD-L1. However, pretreating cancer cells with WCC and ECC to inhibit PD-L1 expression resulted in increased T cell activity and survival rates while also promoting cancer cell death (Figure 3). These findings provide direct evidence that CC extract mitigates immune tolerance and enhances anticancer efficacy by reducing PD-L1 expression in cancer cells.

To evaluate the in vivo efficacy of CC extract in targeting the human immune checkpoint, we utilized hPD-1 mice and hPD-L1/MC-38 cells in fully immunocompetent mice. We first assessed in an ex vivo co-culture system before evaluating CC extract efficacy in a humanized mouse model. hPD-L1/MC-38 cells were inoculated into hPD-1 mice to simulate the tumor microenvironment, leading to tumor mass formation. Tumor-infiltrating CD8+ TILs were then isolated and co-cultured with hPD-L1/MC-38 cells. As expected, both WCC and ECC nearly completely downregulated PD-L1 levels in hPD-L1/MC-38 cells. Co-culture with CD8+ TILs resulted in a two- to three-fold reduction in the viability of hPD-L1/MC-38 cells while significantly enhancing T cell activity and cytotoxicity (Figure 4). These findings provide substantial evidence that CC extract can significantly reduce PD-L1 expression across various cancer types, thereby augmenting anticancer immune responses. Furthermore, this evidence elucidates one of the mechanisms of action underlying its anticancer efficacy in an in vivo mouse model. In a mouse model of colon cancer with human PD-1 and PD-L1, WCC administration reduced tumor growth by approximately 70% and elevated intra-tumoral CD8+ T cells, IL-2, and GrB (Figure 5 and Figure 6). During the experimental period, the administration of WCC did not result in any instances of rapid weight loss or behavioral abnormalities. Additionally, autopsy findings indicated no significant changes in the weight or morphology of the organs, thereby confirming the absence of toxicity.

Our analysis of CC extract components revealed that both BG and EA elicit anti-cancer immune responses by inhibiting the interaction between PD-1 and PD-L1 (Figure 7). These findings align with Kim et al., who identified EA as a key component of black raspberries, showing its effectiveness in immune checkpoint blockade by inhibiting the PD-1/PD-L1 interaction [34]. While EA reduced PD-L1 expression in the UM-UL3 and T24 cell lines [35], it did not affect IFN-γ-induced PD-L1 expression in our study. By contrast, CA inhibits IFN-γ-induced PD-L1 expression via the p-STAT1-IRF1 pathway, enhancing T cell activity and showing a significant antitumor effect, especially in combination with anti-PD-1 antibodies [36]. In this study, we also observed that CA effectively reduced PD-L1 expression and enhanced cancer cell apoptosis and T cell activity in co-cultures. Therefore, these components function as PD-1/PD-L1 ICIs via multiple mechanisms, enhancing the overall anti-cancer immune efficacy of CC extract.

## 5. Conclusions

This study provides the first evidence that CC extract acts as an ICI by downregulating PD-L1 expression in cancer cells. To support this finding, we established a systematic protocol for the pre-treatment of cancer cells prior to their co-culturing with T cells. Furthermore, this research marks the first demonstration of the substantial anticancer efficacy of CC extract in a humanized PD-1/PD-L1 colon cancer mouse model. These findings provide essential foundational data for the design and execution of clinical studies. To effectively use CC extract in cancer treatment, it is essential to validate its synergistic effects with established colon cancer therapies or as an adjuvant. This study focuses on the efficacy of CC extract in modulating the PD-1/PD-L1 immune checkpoint. To address this limitation, we will investigate the anticancer mechanisms in the tumor microenvironment, analyzing cellular populations with single-cell next-generation sequencing technology.

## Data Availability

The data used to support the findings of this study are included within this article.

## Supplementary Materials

The following supporting information can be downloaded at: https://www.mdpi.com/article/10.3390/nu16244415/s1, Figure S1: Effects of WCC and ECC on cancer cells in the co-culture condition with T cells; Figure S2: Effects of WCC and ECC on anti-cancer activity of CD8+ TIL cell in an ex vivo co-culture condition; Figure S3: Inhibitory effects of WCC administration on hPD-L1/MC-38 tumor growth in humanized PD-1 mice; Figure S4: Effects of WCC administration on the tumor infiltrated T cell activity; Figure S5: Effects of compounds in CC extract on anti-cancer activity under co-culture conditions with T cells.
