# Supplementary material for: Caryophylli Cortex Suppress PD-L1 Expression in Cancer Cells and Potentiates Anti-Tumor Immunity in a Humanized PD-1/PD-L1 Knock-In MC-38 Colon Cancer Mouse Model
Source: Nutrients. 2024 Dec 23;16(24):4415. doi: 10.3390/nu16244415 (PMC11679492; doi:10.3390/nu16244415)
Supplement: Supplementary file 1 [file nutrients-16-04415-s001.zip › nutrients-3370787-supplementary.pdf]

Supplementary Materials for

Caryophylli Cortex suppress PD-L1 expression on cancer cells and potentiates anti-tumor immunity in the humanized PD-1/PD-L1 knock-in tumor model

Aeyung Kim[1,*], Eun-Ji Lee[1], Jung Ho Han[1] and Hwan-Suck Chung[1,*]

[1] Korean Medicine (KM) Application Center, Korea Institute of Oriental Medicine, Daegu 41062, Republic of Korea

* Correspondence to:

A. Kim, KM Application Center, Korea Institute of Oriental Medicine, Daegu 41062, Republic of Korea. Tel.: +82-53-940-3830, E-mail: aykim71@kiom.re.kr

H-S. Chung, KM Application Center, Korea Institute of Oriental Medicine, Daegu 41062, Republic of Korea. Tel.: +82-53-940-3875, E-mail: hschung@kiom.re.kr

This file includes: Figure S1-S5

Supplementary Figure S1

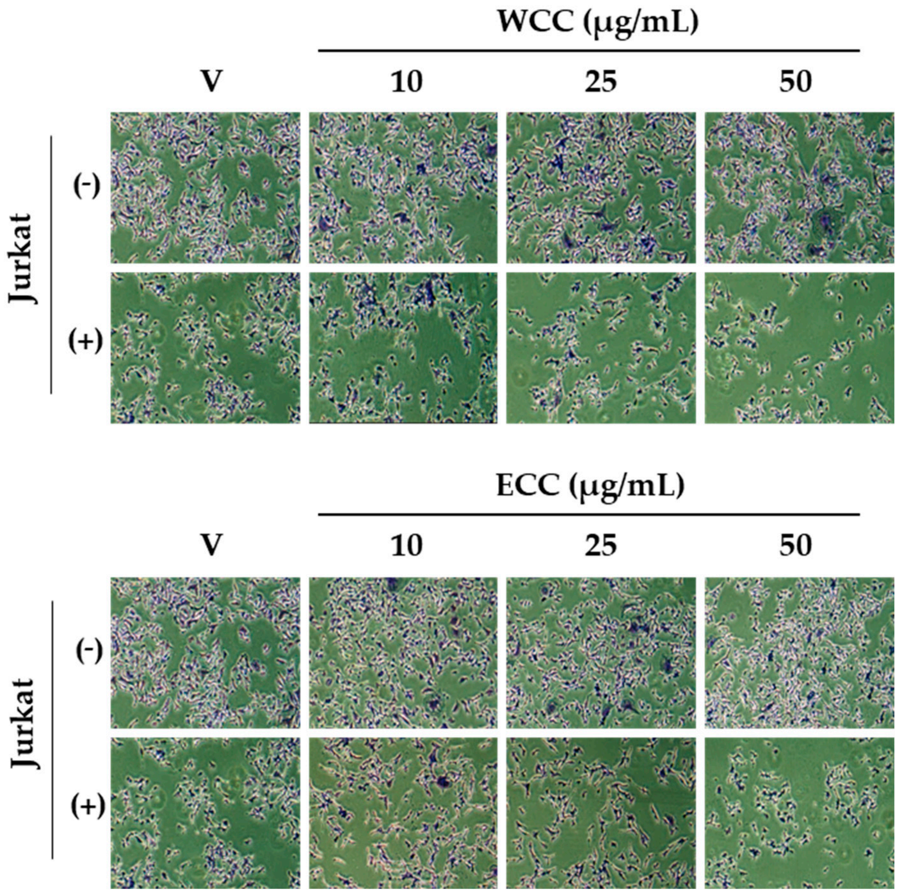

Figure S1. Effects of WCC and ECC on cancer cells in the co-culture condition with T cells. MDA-MB231 cells were pretreated with indicated concentrations of WCC and ECC for 24 h, then co-cultured with or without Jurkat cells for another 24 h. After washing out Jurkat cells, the remaining cancer cells were stained with crystal violet solution and observed under an inverted microscope.

Supplementary Figure S2

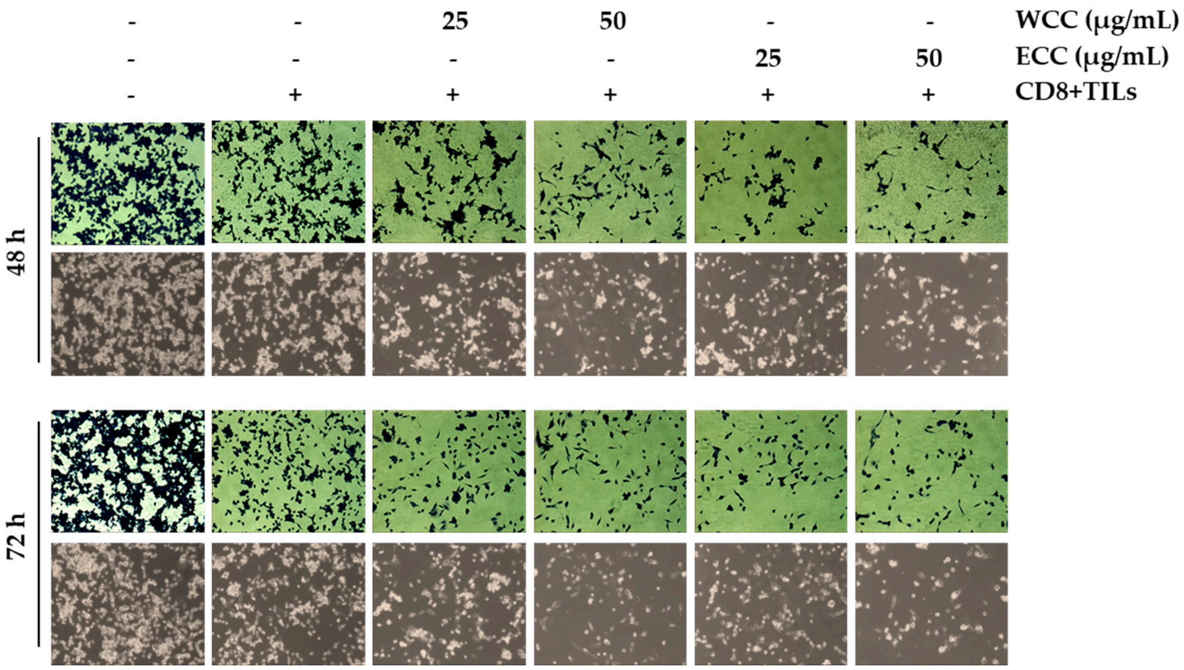

Figure S2. Effects of WCC and ECC on anti-cancer activity of CD8+TIL cell in an *ex vivo* co-culture condition. hPD-L1/MC-38 cells were pretreated with indicated concentrations of WCC and ECC for 24 h, then co-cultured with or without CD8+TIL cells for another 48-72 h. After washing out CD8+TIL cells, the remaining hPD-L1/MC38 cells were stained with crystal violet solution and observed under an inverted microscope.

Supplementary Figure S3

A

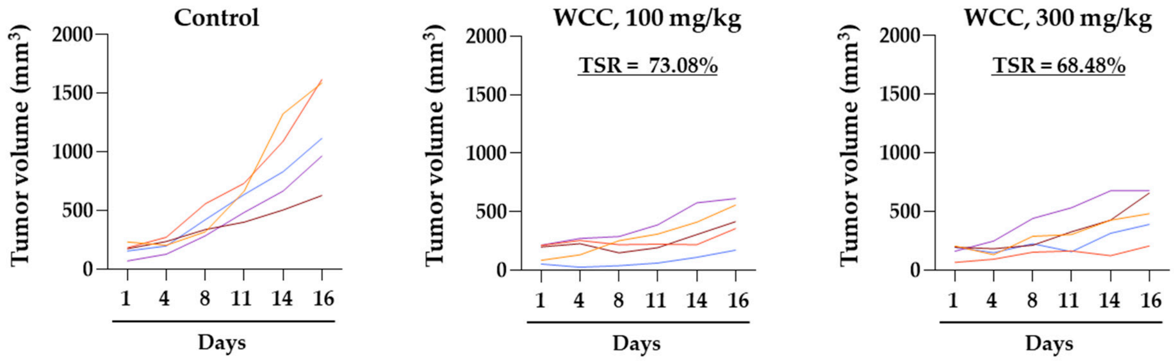

B

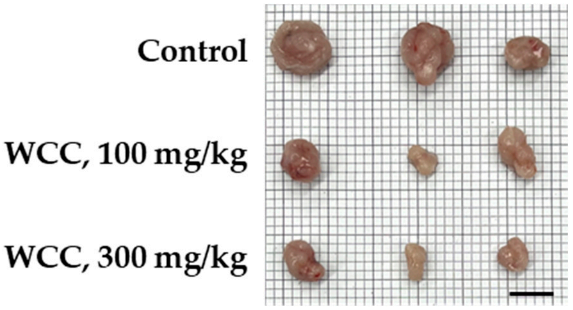

Figure S3. Inhibitory effects of WCC administration on hPD-L1/MC-38 tumor growth in humanized PD-1 mice. (A) hPD-L1/MC38 cells were inoculated subcutaneously into hPD-1 knock-in C57BL6/J mice. Tumor size was measured on days 4, 8, 11, 14, and 16 following the administration of either a vehicle (control) or WCC at doses of 100 or 300 mg/kg. The tumor volumes for individual mice are presented in a plot (n = 5). **(B)** A representative image of the tumor mass excised on day 16 is shown (n = 3). Scale bar = 10 mm.

Supplementary Figure S4

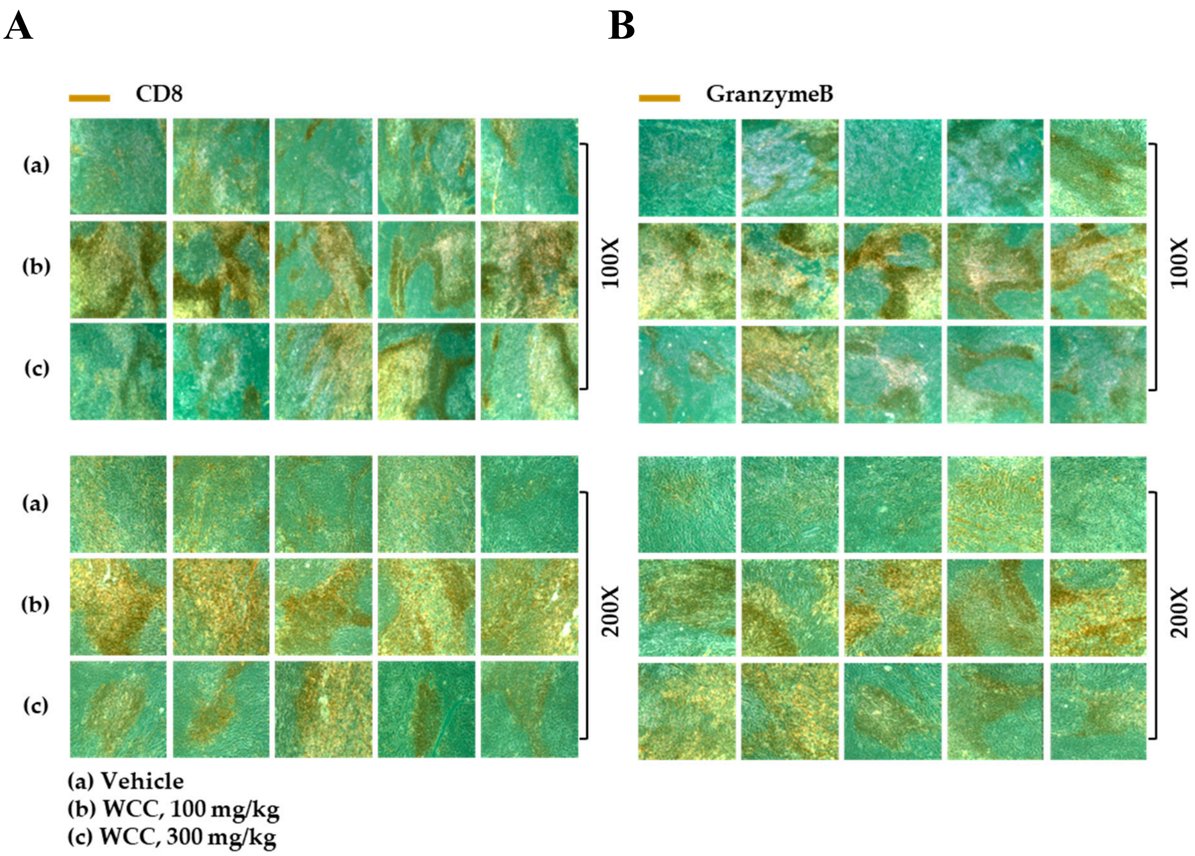

Figure S4. Effects of WCC administration on the tumor infiltrated T cell activity. On day 16, tumor masses were excised from mice that had been administered either a vehicle or WCC at doses of 100 or 300 mg/kg. These samples were subsequently subjected to immunohistochemical staining for CD8 **(A)** and granzyme B **(B)**.

Supplementary Figure S5

A

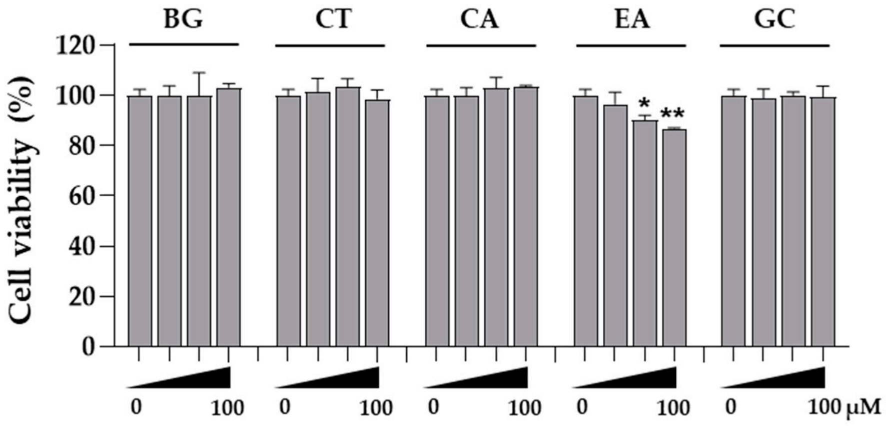

B

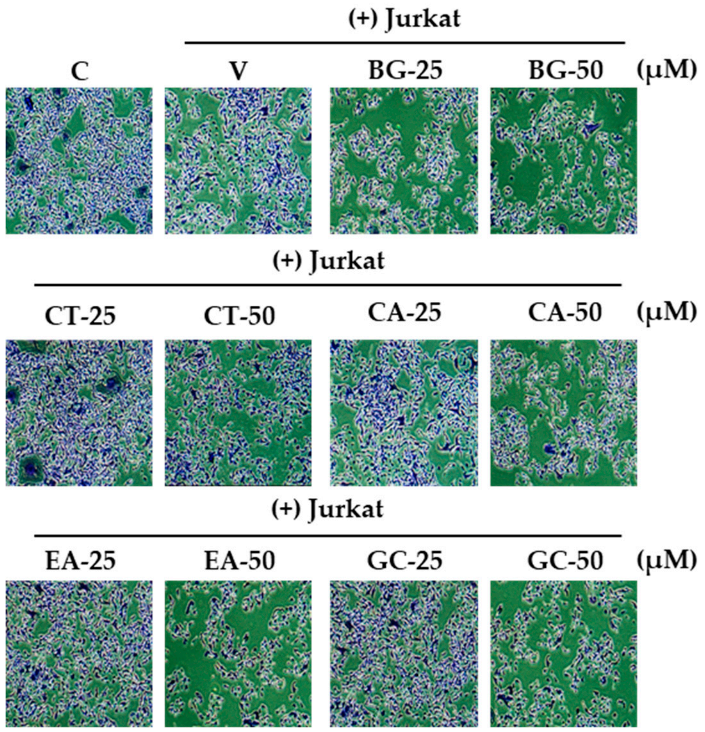

Figure S5. Effects of compounds in CC extract on anti-cancer activity under co-culture conditions with T cells. (A) MDA-MB231 cells were treated with each compound for 24 h to assess cell viability using the CCK assay. **(B)** MDA-MB231 cells were pretreated with each compound for 24 h, then co-cultured with Jurkat cells. After 24 h, surviving cancer cells were examined using crystal violet staining. *$p < 0.05$, **$p < 0.01$ vs. vehicle-treated cells.
